# Effects of vagus nerve stimulation on extinction of conditioned fear and post-traumatic stress disorder symptoms in rats

**DOI:** 10.1038/tp.2017.191

**Published:** 2017-08-22

**Authors:** L J Noble, I J Gonzalez, V B Meruva, K A Callahan, B D Belfort, K R Ramanathan, E Meyers, M P Kilgard, R L Rennaker, C K McIntyre

**Affiliations:** 1 Behavior and Brain Sciences, The University of Texas at Dallas, Richardson, TX, USA

## Abstract

Exposure-based therapies help patients with post-traumatic stress disorder (PTSD) to extinguish conditioned fear of trauma reminders. However, controlled laboratory studies indicate that PTSD patients do not extinguish conditioned fear as well as healthy controls, and exposure therapy has high failure and dropout rates. The present study examined whether vagus nerve stimulation (VNS) augments extinction of conditioned fear and attenuates PTSD-like symptoms in an animal model of PTSD. To model PTSD, rats were subjected to a single prolonged stress (SPS) protocol, which consisted of restraint, forced swim, loss of consciousness, and 1 week of social isolation. Like PTSD patients, rats subjected to SPS show impaired extinction of conditioned fear. The SPS procedure was followed, 1 week later, by auditory fear conditioning (AFC) and extinction. VNS or sham stimulation was administered during half of the extinction days, and was paired with presentations of the conditioned stimulus. One week after completion of extinction training, rats were given a battery of behavioral tests to assess anxiety, arousal and avoidance. Results indicated that rats given SPS 1 week prior to AFC (PTSD model) failed to extinguish the freezing response after eleven consecutive days of extinction. Administration of VNS reversed the extinction impairment and attenuated reinstatement of the conditioned fear response. Delivery of VNS during extinction also eliminated the PTSD-like symptoms, such as anxiety, hyperarousal and social avoidance for more than 1 week after VNS treatment. These results provide evidence that extinction paired with VNS treatment can lead to remission of fear and improvements in PTSD-like symptoms. Taken together, these findings suggest that VNS may be an effective adjunct to exposure therapy for the treatment of PTSD.

## Introduction

Post-traumatic stress disorder (PTSD) affects 22.4 million Americans and can develop following highly stressful experiences, such as combat or sexual assault.^1^ Although most individuals who have traumatic experiences exhibit transient symptoms of stress, ~30% of these individuals suffer from symptoms for longer than 1 month and meet the criteria for diagnosis of PTSD.^1^ According to the fifth edition of the *Diagnostic and Statistical Manual*, an individual may be diagnosed with PTSD after experiencing or witnessing trauma in addition to presenting the following symptoms: re-experiencing the trauma (that is, experiencing emotional or physical distress in response to reminders of the trauma); avoidance of trauma-related stimuli; negative affect, including loss of interest in enjoyable activities; and heightened startle and arousal. Symptoms must persist for more than 1 month and cause significant social or occupational dysfunction.^2^ The prevalence of PTSD is greater in individuals who have experienced multiple traumatic events, suggesting that earlier stressors predispose individuals to the development of PTSD following a traumatic event later in life.^3, 4, 5^

Exposure-based therapies are considered the gold standard of treatment for PTSD.^6^ The goal of exposure-based therapies is to replace conditioned associations of the trauma with new, more appropriate associations. These therapies are based on Pavlov’s observations that learned associations can be modified with extinction training.^7^ Despite their demonstrated therapeutic efficacy, exposure-based therapies for PTSD have high nonresponse and dropout rates.^8, 9, 10^ PTSD patients appear to be resistant to exposure-based therapies because of a generalized extinction deficit.^11, 12, 13, 14^ Further, PTSD patients are impaired in their ability to extinguish conditioned fears that are acquired in controlled laboratory studies.^12, 15, 16^ Adjuvant treatments that improve the consolidation of extinction learning may improve the effectiveness of exposure-based therapies.

Vagus nerve stimulation (VNS) was approved by the Federal Food and Drug Administration for the prevention of seizures in patients with drug-resistant epilepsy in 1997. Considering that VNS can enhance memory consolidation in rats and humans,^17, 18^ we hypothesized that administration of VNS during extinction training could enhance consolidation of an extinction memory. We recently reported that VNS enhanced consolidation of fear extinction following auditory fear conditioning (AFC) and promoted synaptic plasticity in the brain circuitry underlying extinction memory.^19, 20^ These findings suggest that VNS might also be effective in enhancing extinction memory in a rat model of PTSD. To investigate this possibility, we used the single prolonged stress (SPS) procedure in rats, which models successive traumatic events and increases susceptibility for PTSD-like symptoms following fear conditioning.^21^ Like PTSD patients, rats subjected to SPS and fear conditioning exhibit impaired extinction of conditioned fear;^21, 22^ this impairment is specifically seen during consolidation of extinction.^23^ Rats subjected to SPS show behaviors that can be compared to PTSD symptoms, including re-experiencing the trauma, elevated anxiety, arousal and avoidance.^21, 22, 23, 24^ Here we investigated the effects of pairing VNS with exposure to the conditioned stimulus (CS) during extinction on the conditioned fear response and on other PTSD-like symptoms in rats subjected to SPS. The findings suggest that VNS enhances extinction and attenuates reinstatement of fear. Furthermore, VNS administration during extinction is associated with a reduction in PTSD-like symptoms 1 week later.

## Materials and methods

### Animals

All procedures were carried out in accordance with the NIH Guide for the Care and Use of Laboratory Animals, and were approved by the Institutional Animal Care and Use Committee of the University of Texas at Dallas. Male Sprague-Dawley rats (Taconic, Hudson, NY, USA) weighing 225–250 g on arrival were housed on a 12-h light/dark cycle (lights on at 0700 hours) with access to food and water *ad libitum*. Only male rats were used, as previous results indicate that female rats are not susceptible to the extinction impairment produced by SPS.^24^ Criteria for exclusion of rats from the analysis was performance ⩾2 s.d. away from the mean on any task.

**Figure 1:**
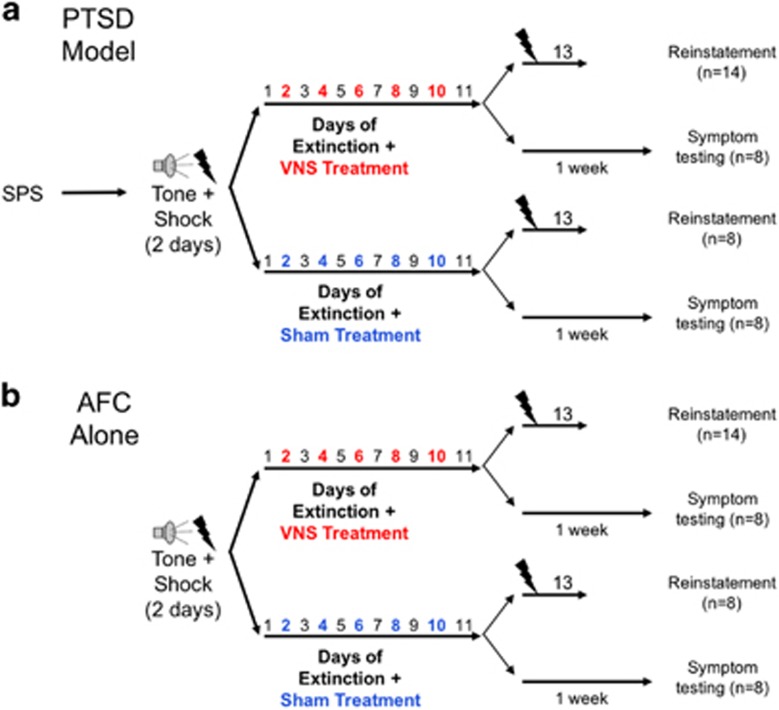
Timeline for extinction, reinstatement and behavioral assays. (**a**) Protocol for PTSD model rats. Rats underwent the SPS procedure followed by 2 days of AFC and 11 days of extinction, five were paired with VNS (red) or sham stimulation (blue) on alternate (even) days. Freezing in the presence of the CS was used as a measure of conditioned fear. Conditioned fear was measured on the odd days that fell between VNS or sham stimulation days. Following extinction, some rats underwent a reinstatement trial where they received one unsignaled footshock. The day after the footshock, these rats were given another day of extinction to measure conditioned fear. The remaining rats were tested on a battery of behaviors to measure PTSD symptoms 1 week after the end of extinction. The order of the tests was counterbalanced. (**b**) Protocol for AFC alone rats. Rats in the AFC alone group were treated exactly like PTSD model rats; however, the AFC alone group did not undergo the SPS procedure. AFC, auditory fear conditioning; PTSD, post-traumatic stress disorder; SPS, single prolonged stress; VNS, vagus nerve stimulation.

To investigate the effects of VNS on extinction and reinstatement of conditioned fear, 22 rats were subjected to the SPS procedure (see ‘Rat model of PTSD’ section below),^21^ followed 1 week later by AFC. Rats subjected to the SPS procedure and AFC were referred to as ‘PTSD model’ rats. Extinction training began 24 h after AFC. The full course of extinction consisted of 11 days of exposure to the CS without reinforcement. During extinction, odd numbered days (extinction days 1, 3, 5, 7, 9 and 11) were used as tests of conditioned fear; four presentations of the CS were administered and conditioned fear to the CS was measured. On even numbered days (extinction days 2, 4, 6, 8 and 10), VNS or sham stimulation was administered and temporally paired with the four CS presentations. Fourteen of the PTSD model rats were given VNS during extinction, and eight were given sham stimulation (Figure 1a).

Twenty-four rats were given the same AFC and extinction without the SPS procedure. These rats were referred to as ‘AFC alone’ rats. Of these, 14 were given VNS during extinction, and 8 were given sham stimulation during extinction (Figure 1b). Twenty-four hours after extinction day 11, rats were given a single reminder footshock. Reinstatement was tested the following day by measuring freezing in response to the conditioned cue.

To test the effects of VNS on PTSD-like symptoms, separate rats were given AFC with SPS (PTSD model rats, *n*=16) or without SPS (AFC alone rats, *n*=16) and exposed to the same 11 days of extinction. Eight rats from each group were given VNS during extinction and eight were given sham stimulation. Seven to ten days after completion of all 11 extinction days, rats were tested on a battery of behavioral tests to examine generalized anxiety, arousal, avoidance and social interaction (Figure 1). The order of test administration was counterbalanced to control for potential interactions. These rats were not given a reminder shock or reinstatement test.

### Cuff electrode preparation

Cuff electrodes were prepared as previously described.^25^ In brief, platinum–iridium wire electrodes were affixed to biocompatible micro-renathane cuffs (1.25 mm inner diameter, 2.5 mm outer diameter, 4.0 mm long). Omnetics four-pin connectors were used to connect the VNS cuff to an AM systems stimulator. Two of the connector pins made contact with the platinum–iridium wires in the cuff in order to deliver stimulation to the vagus nerve.

### Surgical implantation of cuff electrode

Surgery protocols are described in detail elsewhere.^19, 20, 25^ In brief, rats were anesthetized with isoflurane (2% at an oxygen flow rate of 600–800 ml/min). The left vagus nerve was located at the cervical level and isolated from other tissue. The left vagus nerve was selected to avoid stimulation effects on the sinoatrial node. Central activation from the left vagus nerve is bilateral.^26^ The cuff was placed around the nerve and secured in place with a suture. The platinum–iridium wires were tunneled subcutaneously behind the ear to the top of the head and connected to the omnetics connector, which was affixed to the skull using acrylic, to make the headcap. Cessation of breathing was used to test for correct implantation and effectiveness of the VNS cuff; following implantation, while under anesthesia, current (0.8 mA, 1 s) was applied through the cuff to test for cessation of breathing. If cessation of breathing was not observed, the cuff was adjusted or replaced. For sham rats, surgery was conducted in the same manner to isolate the vagus nerve, but the rats were not implanted with a cuff. Animals were given 1 week to recover following surgery.

### Rat model of PTSD

Procedures for SPS were adapted from methods developed by Liberzon *et al.*^21^ In brief, rats were restrained for 2 h in a plastic cone. Immediately after restraint, rats were forced to swim in a tank of water (22.0-inch diameter, 20 °C) for 20 min. Following a 15-min recuperation period, rats were placed in a desiccator and exposed to diethyl ether vapor (Sigma, St. Louis, MO, USA) until they became anesthetized and unresponsive. They were immediately returned to their home cages and left socially isolated for 1 week.

### AFC

On the first day of AFC, rats were exposed to four pretones (four 9 kHz tones, lasting 30 s, at an intensity of 70 dB, administered without any reinforcement) to assess baseline freezing to the tone. Immediately after the pretones, rats received eight tones coupled with a footshock (1 s, 0.4 mA). The tones were presented at a random inter-stimulus interval of between 120 and 240 s. Each shock was administered at a randomized time during the last 25 s of the 30 s tone presentation. Twenty-four hours later, rats underwent a second day of AFC consisting of eight more tone-shock pairings administered in the same way as the previous day, excluding the pretones. All AFC took place in context A (electric grid floor, no olfactory cue). To compare acquisition of conditioned fear between AFC alone rats and PTSD model rats, sessions were recorded and scored by two researchers blind to the treatment conditions. Freezing during the tone, defined as the cessation of movement aside from breathing,^27^ was used as a measure of conditioned fear. We chose this AFC protocol to compare findings in the PTSD model to what we have observed in normal rats using a similar protocol. In this previous study, we found that eight tone-shock pairings per day for 2 days, with unpredictable shock timing during the 30 s tone produced conditioned fear that was not fully extinguished after 11 days of extinction in sham-treated controls.^19^

### Extinction days

Twenty-four hours after both days of AFC, rats underwent 11 days of extinction in context B. Context B consisted of the same conditioning chamber, but contained a distinct plexiglas insert to change the texture of the floor and the addition of an odor (peppermint oil). Each day of extinction consisted of four presentations of the CS (tone) in the absence of any reinforcement (shock). Based on evidence that VNS can enhance memory consolidation, extinction was carried out over several days to allow for consolidation of the extinction memory. This extended protocol for extinction more closely resembles a clinical timeline for individuals with PTSD who would undergo multiple days of exposure-based therapies. Two observers who were blind to the treatment groups measured the percent of time spent freezing during each 30-s tone, which was recorded as the measure of conditioned fear. Freezing times below 10% of the total 120 s of tone exposure was considered remission of fear.^19^ During extinction, rats initially respond to being connected to the stimulator, this occurs in both sham-treated and VNS-treated rats. This response is typically present only during the first extinction session. To avoid potential performance effects of VNS or sham stimulation, conditioned fear responses were measured only on alternate, non-stimulation days (extinction days 1, 3, 5, 7, 9 and 11) when the rats were not connected to the stimulator (Figure 1). Although we have not systematically measured unintended effects of VNS, we have noticed that both sham- and VNS-treated rats occasionally attend to the connector after it is attached to the headcap. This variable behavior could interfere with the conditioned response. In addition, measuring conditioned fear on days when rats were not receiving VNS or sham stimulation made it less likely that interoceptive state-dependent effects of VNS could serve as a safety signal, and provided an opportunity to observe the effect of VNS on consolidation of extinction memory.

### VNS and sham stimulation

Treatment with VNS or sham stimulation was given during extinction on even numbered days (extinction days 2, 4, 6, 8 and 10). To administer stimulation, an AM systems stimulator was connected to the cuff connector on the headcap via a 25.0 cm long PVS multiconductor cable (Cooner Wire, Los Angeles, CA, USA). Stimulation started 150.0 ms before the onset of each tone and then continued for the duration of the tone. VNS was given at a frequency of 20Hz, an intensity of 0.4 mA for 30 s with a 100 μs pulse width. Sham-treated rats were connected to the stimulator in the same way as VNS-treated rats, but did not receive stimulation.

### Reinstatement

Following the completion of extinction, reinstatement of conditioned fear was tested. Twenty-four hours after the 11th day of extinction, rats (*N*=44) were placed in context A and given one unsignaled footshock (unconditioned stimulus) delivered for 1 s at 0.4 mA intensity, in the absence of the tone (CS). Rats remained in context A for 5 min after the footshock. To observe the reaction to the reinstatement shock, sessions were recorded and scored by two researchers blind to the treatment conditions. Freezing was recorded during the entire 5-min observation period. Twenty-four hours after administration of the unconditioned stimulus, rats were exposed to the CS in context B, to test for reinstatement of fear.

### Elevated plus maze

To test generalized anxiety, rats were placed on the central part of an elevated plus-shaped maze (10.0 cm wide, 50.0 cm long, 55.0 cm off the floor) with walls (30.0 cm tall) on two opposing arms and no walls on the other opposing arms. During a 10-min test, time spent in the open arms, time spent in the closed arms and time spent in the center of the maze were recorded. Rats were considered to be in an arm when all four paws were in that arm at one time. All behavior was recorded and scored by two blind researchers. Time spent in the open arms and entries into the open arms were taken as a measure of risk taking.^28^ Percent of total time spent moving was taken as a control measure of general locomotion.

### Acoustic startle response

Before startle behaviors were measured, rats were placed into the apparatus for 5 min to habituate them to the cage. On the following day, rats were placed into the same 20 × 20 × 20 cm^3^ wire-mesh cage centered on a startle platform (Lafayette Instrument, Lafayette, IN, USA) that used a piezoelectric transducer to generate a continuous record of the rat’s activity. Startle responses were elicited by 50.0 ms bursts of white noise at 95 dB sound pressure level. Each rat was subjected to 20 presentations of the startle stimulus with an inter-stimulus interval of 180 s. The waveform of each response served as the measure of the startle response.

### Marble burying

To test novel object avoidance, rats underwent a marble burying task. Rats exposed to a noxious object in their homecage will vigorously bury that object, a phenomenon known as defensive burying.^29^ This avoidance behavior can be seen in rats following fear conditioning with non-salient, novel objects.^30^ This defensive burying of novel objects is sensitive to anxiolytic treatments and is used to measure anxiety and avoidance behavior.^31^ Following habituation to the novel bedding, rats were individually placed into a cage that was identical to their homecage with BioFresh nitrocellulose comfort bedding (3.0 cm deep). Fifteen identical, shiny marbles were placed in three rows in the rear third of the cage. After 10 min, the number of marbles buried was counted and recorded as a measure of novel stimulus avoidance. Only marbles that were more than 2/3 covered by bedding were considered buried. Percent of marbles buried = (number marbles buried/number of marbles present) × 100.

### Social interaction

A three-chamber social interaction task was used to assess social behaviors. The apparatus consisted of three equal-sized chambers: the nonsocial zone, the social zone and the center. The nonsocial zone contained a small wire cage that was empty and sealed; the social zone contained an identical wire cage with a stimulus rat inside. The stimulus rat was matched in size and sex to the experimental rat. The experimental rat was placed into the center of the apparatus, facing the nonsocial zone, and allowed to explore for 10 min. Interactions of the experimental rat with the stimulus rat, time spent in the nonsocial zone, time spent in the social zone and time spent in the center were recorded. A rat was considered to be in a zone of the apparatus only when all four paws were in that zone at once. All behavior was recorded and scored by two experimenters who were blind to treatment conditions. The sociability index (time spent in the social zone – time spent in the nonsocial zone)/(time spent in the social zone + time spent in the nonsocial zone) was used to indicate a preference to interact with or avoid the stimulus rat.

### Data analysis

Data were analyzed using a two-way repeated measures ANOVA or a one-way ANOVA, with a Greenhouse-Geisser correction followed by a Tukey’s *post hoc* test for multiple comparisons or a Holm–Bonferroni sequential correction test for non-independent samples. Statistically significant effects were defined as those with *P-*values that were <0.05. All error bars represent standard error of the mean.

## Results

### VNS administration during exposure to the conditioned stimulus enhanced extinction and reduced reinstatement of conditioned fear

**Figure 2:**
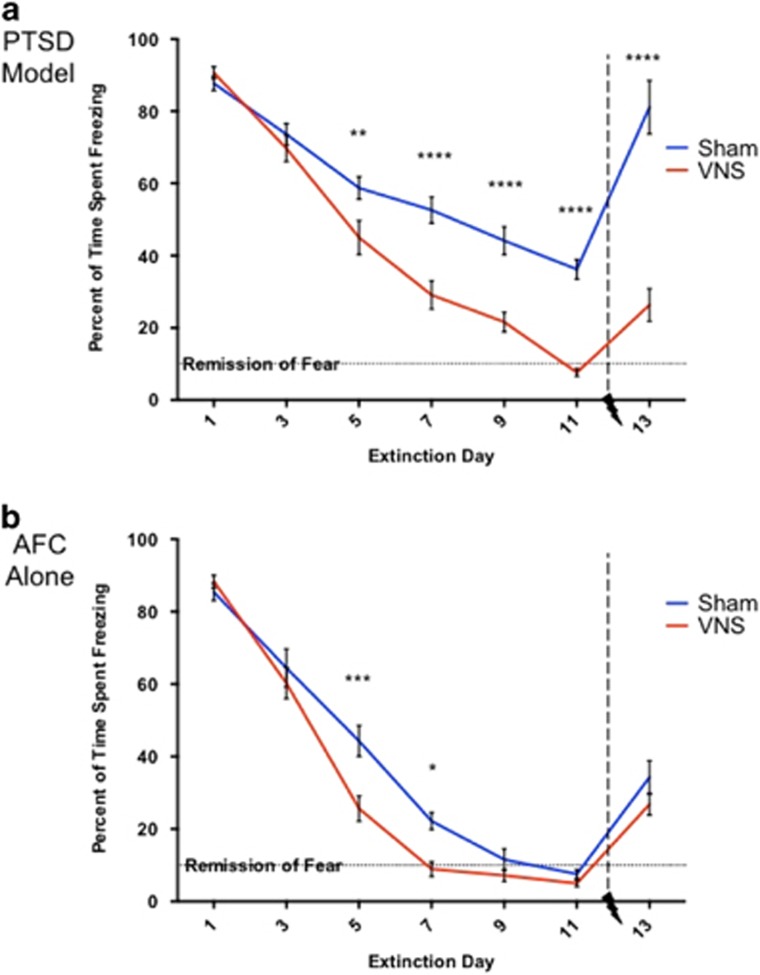
Conditioned fear responding across extinction days. (**a**) VNS treatment reverses maladaptive fear seen in PTSD model rats. Following 11 consecutive days of extinction, rats exposed to the SPS procedure 1 week before AFC (PTSD model rats) did not reach remission of fear. A single, unpaired reminder of the unconditioned stimulus was sufficient to reinstate conditioned fear to the level of freezing measured on the first day of extinction in the PTSD model. PTSD model rats given VNS reached remission of fear by the end of treatment. VNS also attenuated the reinstatement of conditioned fear. Freezing in rats given VNS was significantly reduced compared with freezing on the first day of extinction. PTSD model rats given VNS showed significantly less fear than sham-treated rats on extinction day 5 (**P<0.01) and extinction days 7, 9 and 11 (****P<0.0001). PTSD model rats given VNS showed reduced reinstatement of conditioned fear versus sham-treated rats (****P<0.0001). (**b**) VNS treatment accelerates extinction in control rats subjected to AFC alone. All rats that underwent AFC alone reached remission of fear by the end of extinction. VNS led to more rapid remission of fear than sham stimulation. VNS-treated rats showed reduced freezing versus sham-treated rats on extinction day 5 (***P<0.001) and extinction day 7 (*P<0.05). AFC alone rats alone did not show complete reinstatement of conditioned fear following a reminder of the unconditioned stimulus. AFC, auditory fear conditioning; PTSD, post-traumatic stress disorder; SPS, single prolonged stress; VNS, vagus nerve stimulation.

We modeled PTSD by combining SPS with AFC 1 week later. A two-factor repeated measures ANOVA indicated a significant effect of group across days (F(18 246)=4.764, *P*<0.0001). Although animals with and without SPS exposure demonstrated comparable levels of conditioned fear following AFC, SPS treatment resulted in significantly higher levels of freezing in response to the CS after 11 consecutive days of extinction (Figure 2a, sham). Without VNS, the PTSD model rats did not reach remission of conditioned fear (<10% freezing to the CS). Administration of VNS treatment during five out of the eleven extinction days led to remission of CS-evoked freezing behavior in PTSD model rats (Figure 2a, VNS). By extinction day 5, PTSD model rats given VNS showed decreased freezing versus rats given sham stimulation (*P*<0.01). This effect continued until the completion of treatment: extinction day 7 (*P*<0.0001); extinction day 9 (*P*<0.0001); and extinction day 11 (*P*<0.0001). This supports the hypothesis that VNS treatment can enhance extinction of conditioned fear in an animal model of PTSD.

A single reminder of the unconditioned stimulus (reinstatement footshock on day 12) was sufficient to increase freezing to the CS when presented 24 h later. In PTSD model rats given sham stimulation, the level of conditioned fear returned to that observed before any extinction; freezing behavior on extinction day 13 was not significantly different from extinction day 1 (*P*>0.05). This result indicates that a single stressor is sufficient to restore strong fear behavior in PTSD model rats despite a long period of extinction. The addition of VNS during extinction prevented the reinstatement of conditioned fear observed in PTSD model rats. Freezing behavior in PTSD model rats given VNS was dramatically reduced on extinction day 13 compared to extinction day 1 (*P*<0.00001) (Figure 2a). This result indicates that VNS during extinction makes PTSD model rats resilient to stress-induced relapse.

To compare the fear demonstrated by the PTSD model rats, we examined fear in rats that underwent AFC in the absence of SPS (AFC alone rats). These rats exhibited remission of fear behavior (⩽10% freezing) and resistance to reinstatement (Figure 2b, sham); freezing behavior in sham-treated rats was dramatically reduced on extinction day 13 compared to extinction day 1 (*P*<0.00001). These results indicate that the stability and degree of fear extinction is substantially different between PTSD model rats and AFC alone rats, as previously reported.^21, 22, 23^

Still, in rats that received AFC alone, VNS during extinction accelerated extinction of conditioned fear (Figure 2b). On average, VNS-treated rats reached remission of fear 2 days earlier than sham-treated rats (*P*<0.01). In rats given AFC alone, VNS treatment reduced freezing versus sham on extinction day 5 (*P*<0.001) and extinction day 7 (*P*<0.05). Freezing following reinstatement was not different between the sham- and VNS-treated rats (*P*>0.05).

**Figure 3:**
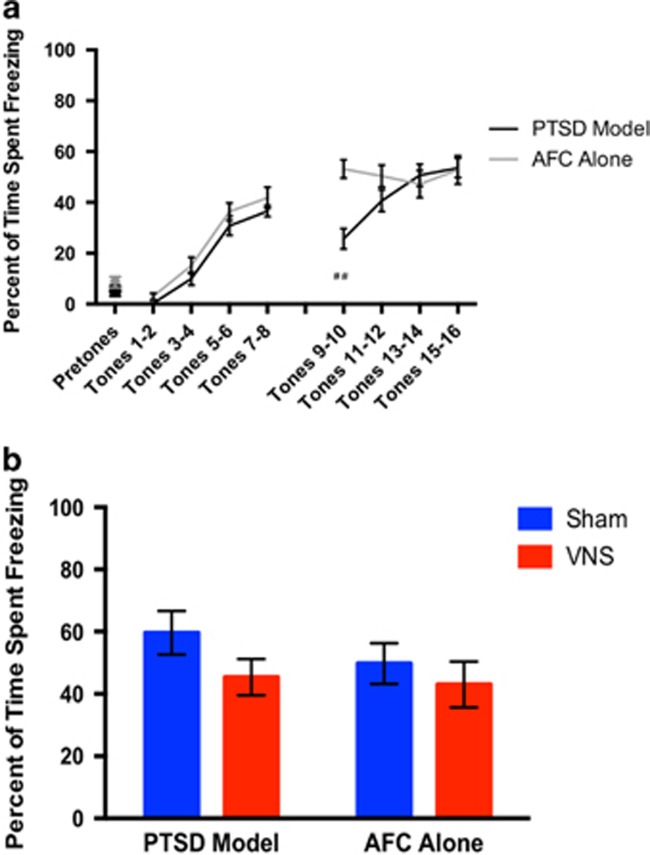
AFC alone rats and PTSD model rats respond similarly to footshock. (**a**) Acquisition of conditioned fear is similar between groups. Prior to VNS- or sham-paired extinction, rats underwent 2 days of AFC. On the first day of AFC, rats were exposed to four pretones to assess baseline freezing to the tone, pretone freezing was similar between groups. Acquisition of conditioned fear was similar between all rats on these days; however, PTSD model rats show a reduction in freezing at tones 9 and 10 versus AFC alone rats (^##^*P*<0.01). This effect is no longer present on subsequent tones. (**b**) Following reinstatement shocks, rats show similar levels of freezing. Following a reinstatement shock in context A, rats from all groups show comparable levels of freezing (*P*>0.05). AFC, auditory fear conditioning; PTSD, post-traumatic stress disorder; VNS, vagus nerve stimulation.

Prior to VNS- or sham-paired extinction, rates of acquisition of conditioned fear between AFC alone rats and PTSD model rats were examined (Figure 3a). A two-factor repeated measures ANOVA indicated a significant effect of group across tones for acquisition of conditioned fear (F(7, 168)= 2.473, *P*<0.05). All rats show a significant effect of time, as levels of freezing increase as the number of tone-shock pairings increases (F(7, 168)=41.36, *P*<0.0001). A Holm–Bonferroni sequential correction revealed a significant difference between PTSD model rats and AFC alone rats only at tones 9 and 10 (*P*<0.01). PTSD model rats showed a deficit in fear retention versus AFC alone rats (*P*<0.01) at the start of the second day of fear conditioning. This could be explained by evidence that SPS impairs consolidation, but has no learning effect within a session.^23^ This effect was not present during other AFC tones or at the start of extinction training (extinction day 1).

A one-way ANOVA revealed no significant effect of freezing following the reinstatement shock (F(2.582, 8.94)=0.522, *P*>0.05). All rats showed similar levels of conditioned fear immediately following reinstatement shock (Figure 3b), indicating the shock was equally aversive to all rats. However, 24 h later, there was a significant difference between PTSD model rats given sham stimulation and all other rats (*P*<0.0001).

A total of two AFC alone rats (1 sham and 1 VNS) met the exclusion criteria. These rats were not included in analysis because they did not exhibit conditioned fear following AFC (freezing behavior was <2 s.d. away from the mean).

### VNS administration during extinction sessions reduced PTSD-like symptoms

#### EPM

**Figure 4:**
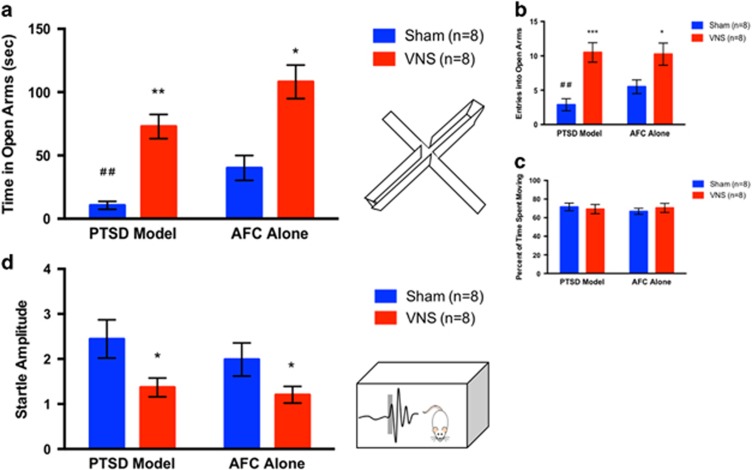
VNS treatment reversed PTSD-like symptoms of anxiety and exaggerated startle. (**a**) VNS administration during extinction reduced anxiety. One week after the completion of extinction, PTSD model rats spent less time in the open arms of the maze than AFC alone rats (^##^*P*<0.01), indicating heightened anxiety. VNS during extinction reversed this effect in PTSD model rats and also decreased anxiety in AFC alone rats. VNS-treated rats spent more time in the open arms versus sham-treated rats in the PTSD model (**P<0.01) and in the control group that underwent AFC alone (*P<0.05). (**b**) VNS treatment increased entries into the open arms. Similar to time spent in the open arms, PTSD model rats showed a reduced number of entries into the open arms versus AFC alone rats (^##^*P*<0.01). Administration of VNS during extinction reversed this effect and increased entries into the open arms in PTSD model rats (***P<0.001) and in AFC alone rats (*P<0.05). (**c**) Total locomotion was not affected. Total time spent moving in the EPM was similar between groups (*P*>0.05). (**d**) VNS treatment reduced startle responses. Following extinction, there was no difference in PTSD model rats versus AFC alone rats. Administration of VNS during extinction reduced startle amplitudes in PTSD model rats versus sham (*P<0.05) and in rats that underwent AFC alone versus sham (*P<0.05). AFC, auditory fear conditioning; EPM, elevated plus maze; PTSD, post-traumatic stress disorder; VNS, vagus nerve stimulation.

To test the effect of VNS on general anxiety, rats were tested on the elevated plus maze (EPM) (Figures 4a and b). A one-way ANOVA revealed a significant effect across groups (F(2.430, 17.01)=17.26, *P*<0.0001). PTSD model rats given sham stimulation spent less time in the open arms than AFC alone rats given sham stimulation (*P*<0.05), and made fewer entries into the open arms (*P*<0.01), indicating that anxiety was elevated in the PTSD model rats. VNS treatment reversed this effect, in that PTSD model rats given VNS during the extinction sessions spent more time in the open arms (*P*<0.01), and made more entries into the open arms versus PTSD model rats given sham stimulation (*P*<0.001). PTSD model rats given VNS during extinction sessions spent a similar amount of time in the open arms as AFC alone rats (*P*>0.05). VNS treatment also increased time spent in the open arms (*P*<0.05) and entries into the open arms in AFC alone rats (*P*<0.05). These results demonstrate that VNS treatment during extinction reduced general anxiety 1 week after treatment. Total locomotion was not different in PTSD model rats versus AFC alone rats, and administration of VNS did not affect total locomotion (Figure 4c).

#### Acoustic startle response

To test for hyperarousal, rats underwent an acoustic startle response test. VNS treatment during extinction reduced startle responses in PTSD model rats and in AFC alone rats (Figure 4d). A one-way ANOVA indicated a significant effect across groups (F(1.40, 9.80)=6.980, *P*<0.05). Startle amplitudes prior to habituation (the first 15 startle bursts) were similar in PTSD model rats given sham stimulation and sham-treated AFC alone rats (*P*>0.05). PTSD model rats that received VNS during extinction showed a reduction in startle amplitude versus sham-treated rats (*P*<0.05). VNS also decreased startle amplitude versus sham-treated AFC alone rats (*P*<0.05). These results demonstrate that although SPS did not increase startle responses, VNS was still effective in reducing startle amplitude.

#### Marble burying

**Figure 5:**
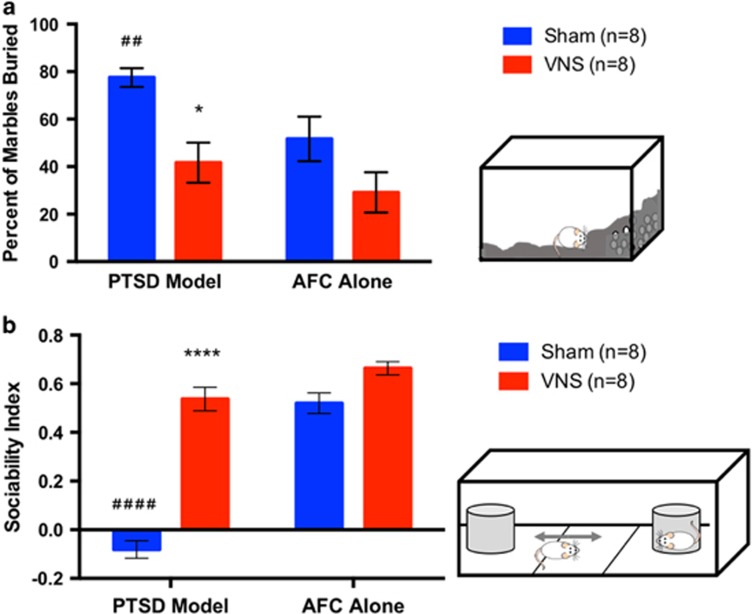
VNS during extinction decreases novel avoidance and social withdrawal behavior. (**a**) VNS reduced novel object avoidance in PTSD model rats. One week following extinction, PTSD model rats showed increased marble burying versus AFC alone rats (^##^*P*<0.01). VNS during extinction reversed this effect PTSD model rats (*P<0.05 versus sham). There was no difference in AFC alone rats. (**b**) VNS increased social interaction. PTSD model rats given sham treatment showed diminished social interaction versus AFC alone rats (^####^*P*<0.0001). Administration of VNS during extinction reversed this effect and increased social interaction (****P<0.0001 versus sham). There was no difference between VNS and sham stimulation in AFC alone rats. AFC, auditory fear conditioning; PTSD, post-traumatic stress disorder; VNS, vagus nerve stimulation.

To test for avoidance of novel objects, rats were tested on a marble burying task. VNS treatment during extinction reduced avoidance in PTSD model rats (Figure 5a). A one-way ANOVA indicated a significant effect across groups F(2.694, 18.86)=6.622, *P*<0.01. PTSD model rats given sham stimulation buried more marbles than AFC alone rats (*P*<0.01). PTSD model rats given VNS during extinction buried fewer marbles than those given sham stimulation (*P*<0.05), and buried a similar number of marbles as AFC alone rats (*P*>0.05). This indicates that VNS treatment during extinction reduced novel avoidance behavior in PTSD model rats.

In AFC alone rats, there was no difference between VNS and sham stimulation (*P*>0.05).

#### Social interaction

To test whether VNS during extinction could reverse abnormal social interactions characteristic of PTSD, rats were evaluated using a social interaction test. PTSD model rats showed social withdrawal. VNS during extinction reversed the social withdrawal and restored normal social behavior (Figure 5b). A one-way ANOVA indicated a significant effect across groups F(2.632, 18.42)=59.44, *P*<0.0001. PTSD model rats given sham stimulation had a negative sociability index, indicating the typical preference to interact with the stimulus rat was deficient. The sociability index for the PTSD model rats was significantly lower than that of AFC alone rats (*P*<0.00001). This was reversed by VNS; PTSD model rats given VNS had a higher sociability index than those given sham stimulation (*P*<0.0001), and the sociability index was not significantly different from that of AFC alone rats. These results show that VNS treatment during extinction improved social interaction in PTSD model rats. There was no significant difference between social interaction indexes of VNS- versus sham stimulation-treated AFC alone rats.

Taken together, these results demonstrate that anxiety-related behavior in PTSD model rats is qualitatively and quantitatively distinct from that of AFC alone rats.

## Discussion

The SPS rat model of PTSD shares important characteristics with PTSD in human subjects. For clinical diagnosis of PTSD, patients must exhibit symptoms from each of the four criteria for more than 1 month.^2^ PTSD patients show extinction impairments that may be responsible for the persistence of fear and anxiety symptoms. Here we observed that exposure to SPS (restraint, swim stress, loss of consciousness and social isolation) 7 days prior to fear conditioning makes rats susceptible to impaired extinction of the fear response. This is consistent with previous observations of extinction impairments in the SPS model.^22, 23^ However, we found that SPS combined with subsequent AFC lead to a fear response to the CS even after 11 days of extinction. PTSD model rats also showed significantly higher reinstatement, a measure of relapse, following a reminder of the unconditioned stimulus. One of the symptom clusters of PTSD is intrusion symptoms, such as distress and re-experiencing after exposure to traumatic reminders. The present findings indicate that the animal model of PTSD demonstrates resistance to extinction learning and re-experiencing the trauma (inferred from the freezing response) in the presence of reminders of the trauma.

Alterations in arousal and reactivity, such as exaggerated startle responses, make up the second symptom cluster. One week after completion of extinction, PTSD model rats showed heightened anxiety on an EPM, but the acoustic startle response was not significantly different in PTSD model rats and AFC alone rats. PTSD patients demonstrate hypervigilance and exaggerated startle responses. It is possible that auditory fear conditioning alone increases the acoustic startle response, obscuring the effect of multiple stressors. For example, the acoustic startle response is potentiated in fear-conditioned rats and humans when it is tested in the presence of conditioned cues.^32, 33^ Avoidance is another symptom cluster that is described in the *Diagnostic and Statistical Manual-5*. PTSD model rats showed an increase in the novel avoidance task of marble burying.

The fourth symptom cluster of PTSD is negative alterations in cognition or mood, such as social withdrawal and persistent negative emotions. Social interaction scores were significantly lower for PTSD model rats than they were for AFC alone rats. In fact, AFC alone rats showed a strong preference for the social zone in the social interaction test, whereas PTSD model rats did not show a preference at all for the social zone over the nonsocial zone. These findings provide evidence of social withdrawal and less engagement in normal activities in the PTSD model. Taken together, these findings suggest that the SPS model of PTSD shows many behaviors that resemble PTSD symptoms, and may be useful in the study of the effects of traumatic events on the brain and behavior.

VNS administration during extinction reversed the extinction impairment observed in PTSD model rats, and VNS improved symptoms from each PTSD symptom cluster, including re-experiencing fear, elevated anxiety, arousal, avoidance and social withdrawal. PTSD model rats continued to exhibit a freezing response to the CS after 11 consecutive days of extinction. Others have shown enhancement in conditioned fear following contextual fear conditioning in the SPS model.^34^ The extinction impairment in PTSD model rats reported here cannot be explained by a conditioning enhancement, as PTSD model rats do not show an enhancement in conditioning, in fact they show a temporary deficit in fear retention during tones 9 and 10. VNS administration during extinction reversed the extinction impairment in the rat PTSD model. Like AFC alone rats, PTSD model rats given VNS during extinction demonstrated remission of conditioned fear. This reduction in conditioned fear was also observed following reinstatement. A single reminder of the unconditioned stimulus was sufficient to fully reinstate conditioned fear in PTSD model rats, but VNS treatment during extinction prevented this relapse. These findings suggest that VNS may facilitate progress in exposure therapy by enhancing extinction of conditioned fear and reducing relapse.

Although persistent fear in the presence of reminders of the trauma is a well-recognized PTSD symptom, generalized anxiety, hyperarousal and avoidance behaviors can also be disabling. The administration of VNS during extinction reduced anxiety and avoidance behavior 1 week later on tasks that did not involve the CS. The observation that VNS treatment reduced avoidance of novel stimuli and startle responses, and increased exploratory and social behavior in PTSD model rats suggests that this adjuvant therapy can improve pathological behaviors that are not directly related to specific trauma cues.

Extinction is the goal of exposure-based therapies and VNS enhances extinction. However, the mechanisms by which VNS enhances extinction are not yet known. VNS enhances memory consolidation^17, 18, 19^ and alters the release of neuromodulators into the brain that may promote experience-dependent plasticity.^20, 35, 36, 37, 38, 39^ Pairing VNS with an auditory stimulus alters auditory cortical maps while pairing VNS with motor learning modulates maps in the motor cortex, indicating that the nature of the plasticity is driven by the training that is paired with VNS.^40, 41, 42, 43, 44^ We recently reported that VNS promotes plasticity in the pathway from the infralimbic area of the prefrontal cortex to the basolateral complex of the amygdala in rats.^20, 45^ Humans with PTSD exhibit reduced activation of the ventromedial prefrontal cortex and increased activation of the amygdala.^46, 47^ In addition, extinction impairments, like those observed in rats exposed to SPS, are associated with decreased prefrontal cortical control over amygdala activity.^48, 49^ VNS enhancement of consolidation of the extinction memory via facilitation of plasticity in this circuitry could be responsible for successful extinction following VNS in PTSD model rats.

VNS may also enhance extinction by inhibiting activity of the sympathetic nervous system.^50, 51^ The vagus nerve is sometimes referred to as the ‘vagal brake’ as activation of the vagus nerve activates the parasympathetic system and slows heart rate following the sympathetic stress response.^52^ One study showed that chronic VNS reduced a measure of anxiety in rats,^53^ and another suggested that chronic VNS improved scores on the Hamilton Anxiety Scale in human patients suffering from treatment-resistant depression.^54^ It is possible that an immediate VNS-induced reduction in anxiety contributes to VNS-driven extinction by interfering with the sympathetic response to the CS, thus breaking the association of the CS with fear. In addition, a total of 20 trains of VNS administered over the course of 11 days may be sufficient to produce lasting anxiolytic effects, as has been observed following chronic VNS. Such a long-lasting anxiolytic effect would explain the reduction of general PTSD-like symptoms in VNS-treated rats. However, it is not likely that a general and lasting anxiolytic effect is responsible for VNS-driven remission of fear as unpaired administration of VNS did not enhance extinction of conditioned fear in a previously reported study.^19^

These findings demonstrate that VNS treatment can reverse extinction impairments and provide benefits across a variety of symptoms in a rat model of PTSD. Extinction of conditioned fear in nonhuman animals is frequently used as a preclinical model of exposure therapy.^13, 55, 56, 57^ The present findings suggest that VNS may be an effective adjunct to exposure therapy. Since VNS has been used in tens of thousands of patients with drug-resistant epilepsy^58^ and delivery during exposure therapy requires considerably less stimulation, VNS may be safely used to enhance extinction in the treatment of PTSD and other disorders that show improvements with exposure-based therapies.
